# Supplementary material for: CMTr cap-adjacent 2′-O-ribose mRNA methyltransferases are required for reward learning and mRNA localization to synapses
Source: Nat Commun. 2022 Mar 8;13:1209. doi: 10.1038/s41467-022-28549-5 (PMC8904806; doi:10.1038/s41467-022-28549-5)
Supplement: Supplementary file 1 — Supplementary Information [file 41467_2022_28549_MOESM1_ESM.pdf]

Supplementary information

CMTr cap-adjacent 2`-*O*-ribose methyltransferases are required for reward learning and mRNA localization to synapses

Irmgard U. Haussmann, Yanying Wu, Mohanakarthik P. Nallasivan, Nathan Archer, Zsuzsanna Bodi, Daniel Hebenstreit, Scott Waddell, Rupert Fray, Matthias Soller*

*Correspondence to m.soller@bham.ac.uk

Supplementary Figures

Supplementary Figure 1

Supplementary Figure 2

Supplementary Figure 3

Supplementary Figure 4

Supplementary Figure 5

Supplementary Figure 6

Supplementary Figure 7

Supplementary Figure 8

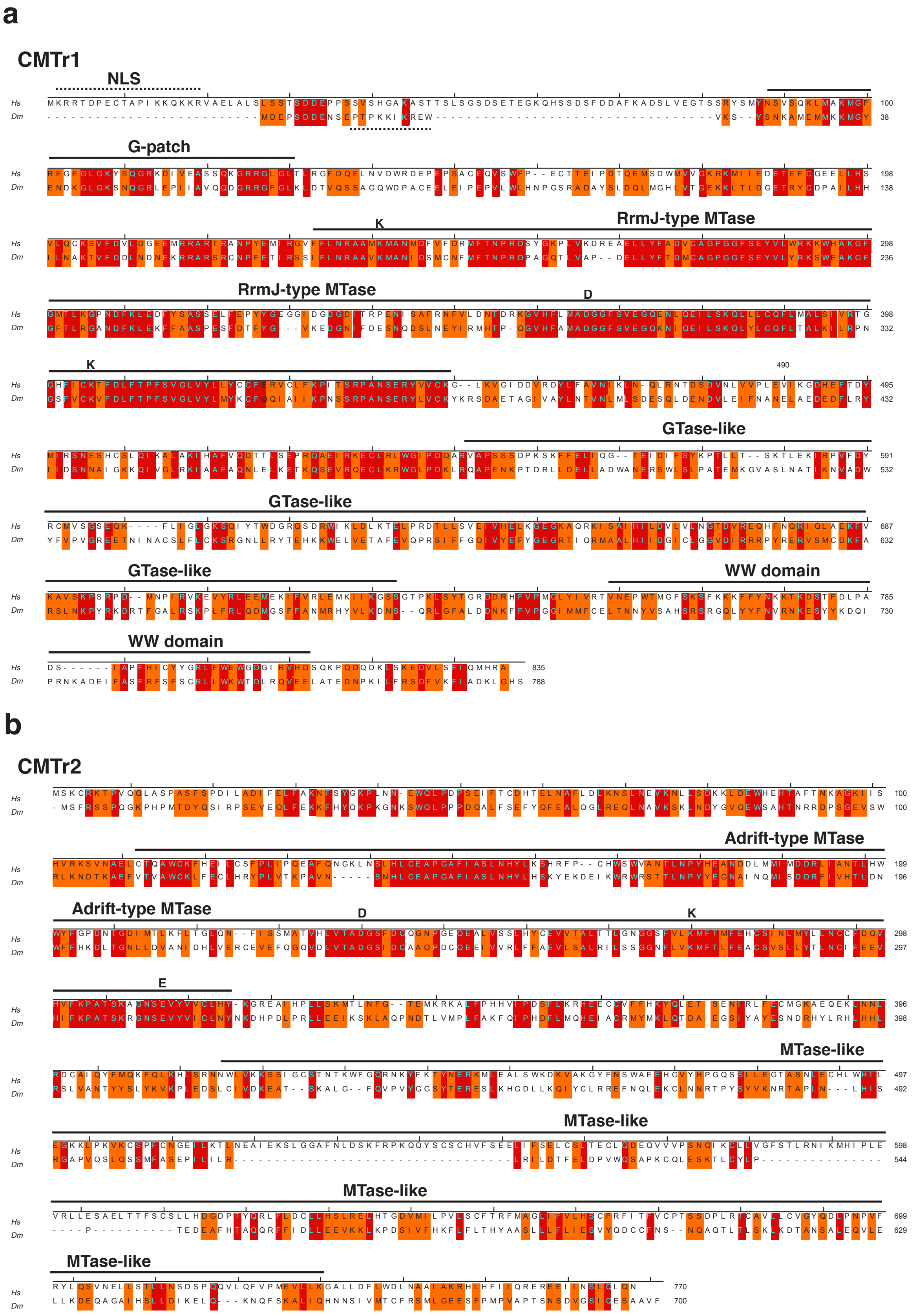

Supplementary Figure 1. Alignment of CMTr1 and CMTr2 from human and *Drosophila*. (a and b) Sequences of human (*Hs*) and *Drosophila* (*Dm*) CMTr1 and CMTr2 are aligned indicating protein domains on top as lines. Identical and conserved amino acids are shown in red and orange, respectively. The three key-residues in the catalytic center are indicated by single letters. The non-conserved position of the nuclear localization signal (NLS) in CMTr1 (a) is indicated by a dashed line.

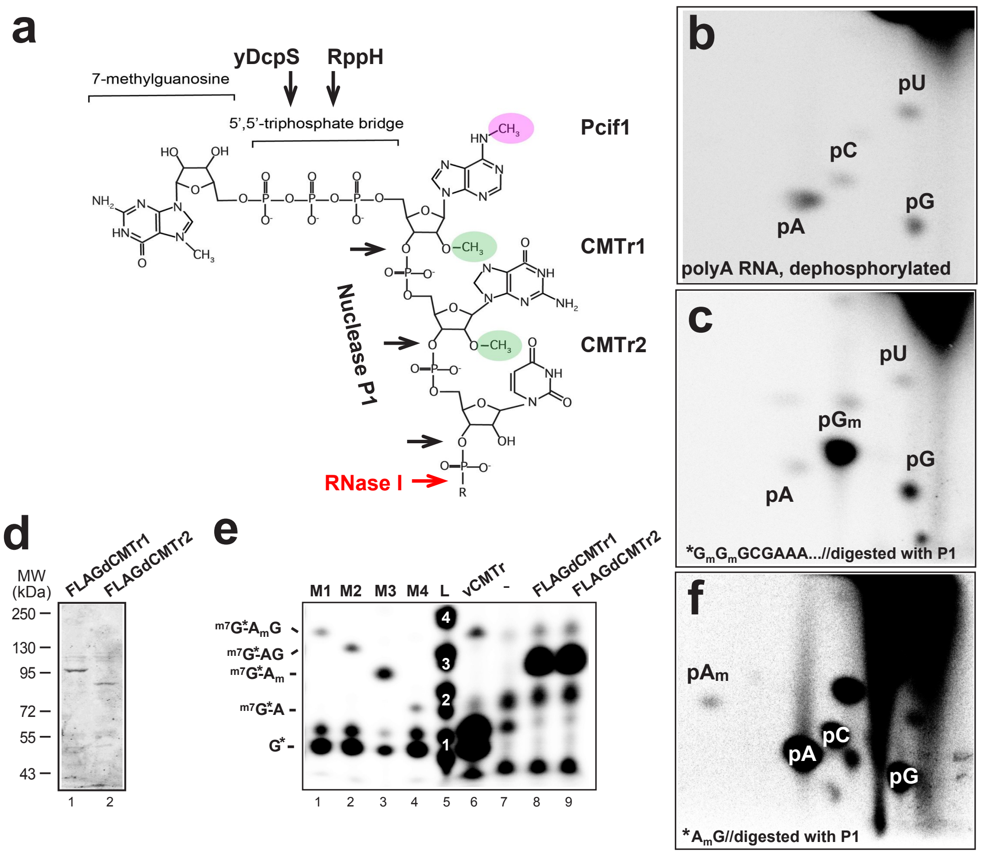

Supplementary Figure 2. Analysis of 5` cap structures.

(**a**) Cap-structure of mRNAs indicating sites of methylation in cap-adjacent nucleotides in green for 2`-*O*-ribose methylation by CMTrs and of adenosine *N6* methylation in pink by Pcif1 in humans. Cleavage site for various decapping enzymes and nucleases are indicated by arrows.

(b) Dephosphorylated and ^{32}P-labeled polyA mRNA reveals little contaminating rRNA and absence of cOMe analyzed by 2D TLC (n=2).

(c) Decapped, dephosphorylated and ^{32}P-labeled *in vitro* transcript starting with GGG previously capped and 2`-*O*-ribose methylated with vaccinia CMTr after digestion with nuclease P1 reveals the position of pGm overlapping with pC on 2D TLC (n=2).

(d) Representative Western-blot from two replicates of FLAG-tagged *Drosophila* CMTr1 and CMTr2 expressed in *Drosophila* S2 cells detected with anti-FLAG antibodies (n=2). Molecular weight markers in kDa are shown on the left.

(e) 2`-*O*-ribose methylation of a $^{32}$PalphaGTP capped *in vitro* transcript by *Drosophila* CMTR1 and CMTr2 expressed in S2 cells, vaccinia CMTr or no CMTr (-). 5`cap structures were separated on a 20% denaturing polyacrylamide gels after digestion with RNAse I (lanes 6-9, right). Markers - M1: RNAse I digested ^{32}PalphaGTP capped *in vitro* transcript starting with AGU and 2`-O-ribose methylated with vaccinia CMTr. M2: RNAse T1 digested $^{32}$PalphaGTP capped *in vitro* transcript starting with AGU. M3: Nuclease PI digested $^{32}$PalphaGTP capped *in vitro* transcript starting with AGU and 2`-O-ribose methylated with vaccinia CMTr. M4: RNAse I digested ^{32}PalphaGTP capped *in vitro* transcript starting with AGU. Sequences of markers are shown on the left and of cap structures from adult flies are shown on the right. L: Single nucleotide ladder with nucleotide number indicated in white.

(f) 2D TLC of *in vitro* 2`-O-ribose methylated ^{32}PalphaGTP capped *in vitro* transcript by *Drosophila* CMTr1 (lane 7 in e), that was decapped, dephosphorylated and ^{32}P-labeled, and then digested with nuclease P1.

Source data for Western blots and gels are provided as a Source Data file.

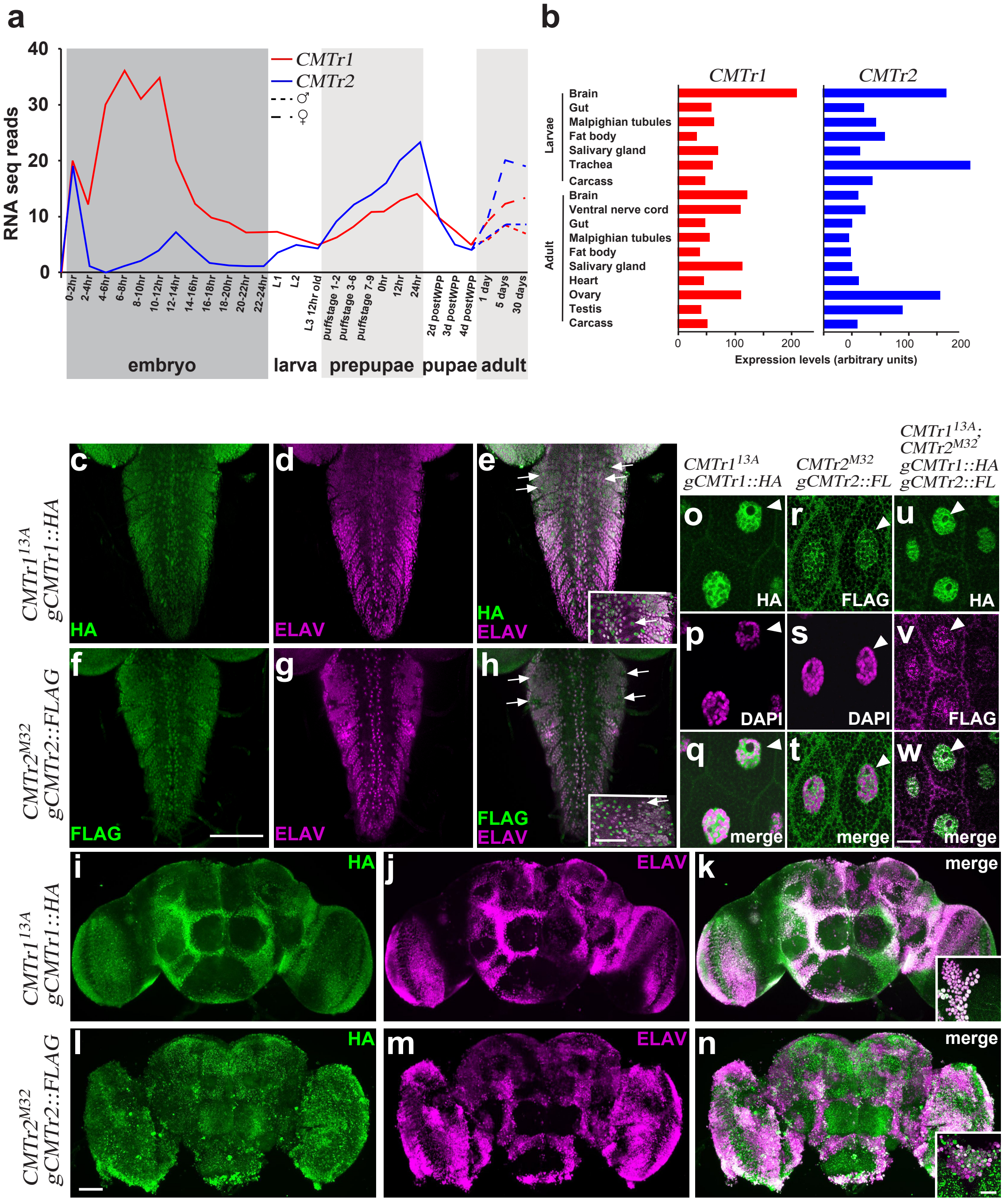

Supplementary Figure 3. Expression of CMTr1 and CMTr2.
(a and b) *CMTr1* and *CMTr2* expression profile during development from RNAseq (a) or in various tissues from microarrays (b) (from flybase.org). **(c-n)** Representative images from six replicates showing expression of CMTr1 and CMTr2 in larval (c-h) or in adult brains (i-n) compared to neuronal marker ELAV. Arrows in e and k point towards expression in non-neuronal cells. Scale bars are 50 μm and 100 μm in F and L, and 10 μm and 5 μm in the inset in H and N. Scale bars are 150 μm. **(o-w)** Representative images from six replicates showing nuclear localization of CMTr1::HA and/or CMTr2::FLAG in salivary glands in mutant background. Arrow heads point towards the nucleolus. Scale bar: 20 μm. Source data for mRNA expression levels are provided as a Source Data file.

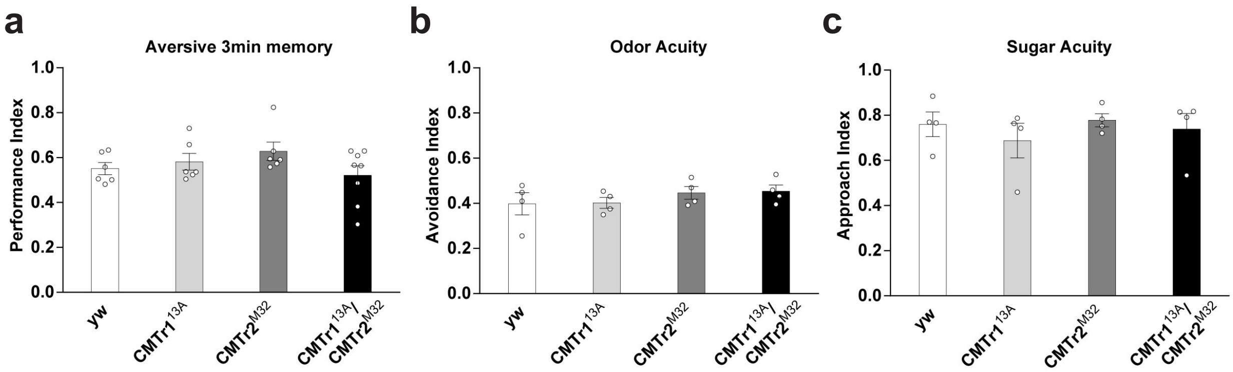

Supplementary Figure 4. Negatively enforced learning and memory, smell and taste are normal in the absence of mRNA cap-adjacent 2`-*O*-ribose methylation. (a-c) Aversive memory immediately after training (*n*=6, except *CMTr1*^{13A}/*2*^{M32} n=8), and odor and sugar acuity (*n*=4) of control, and *CMTr1*^{13A} and *CMTr2*^{M32} single and double mutant flies shown as mean±SE.

Source data for behavioral experiments are provided as a Source Data file.

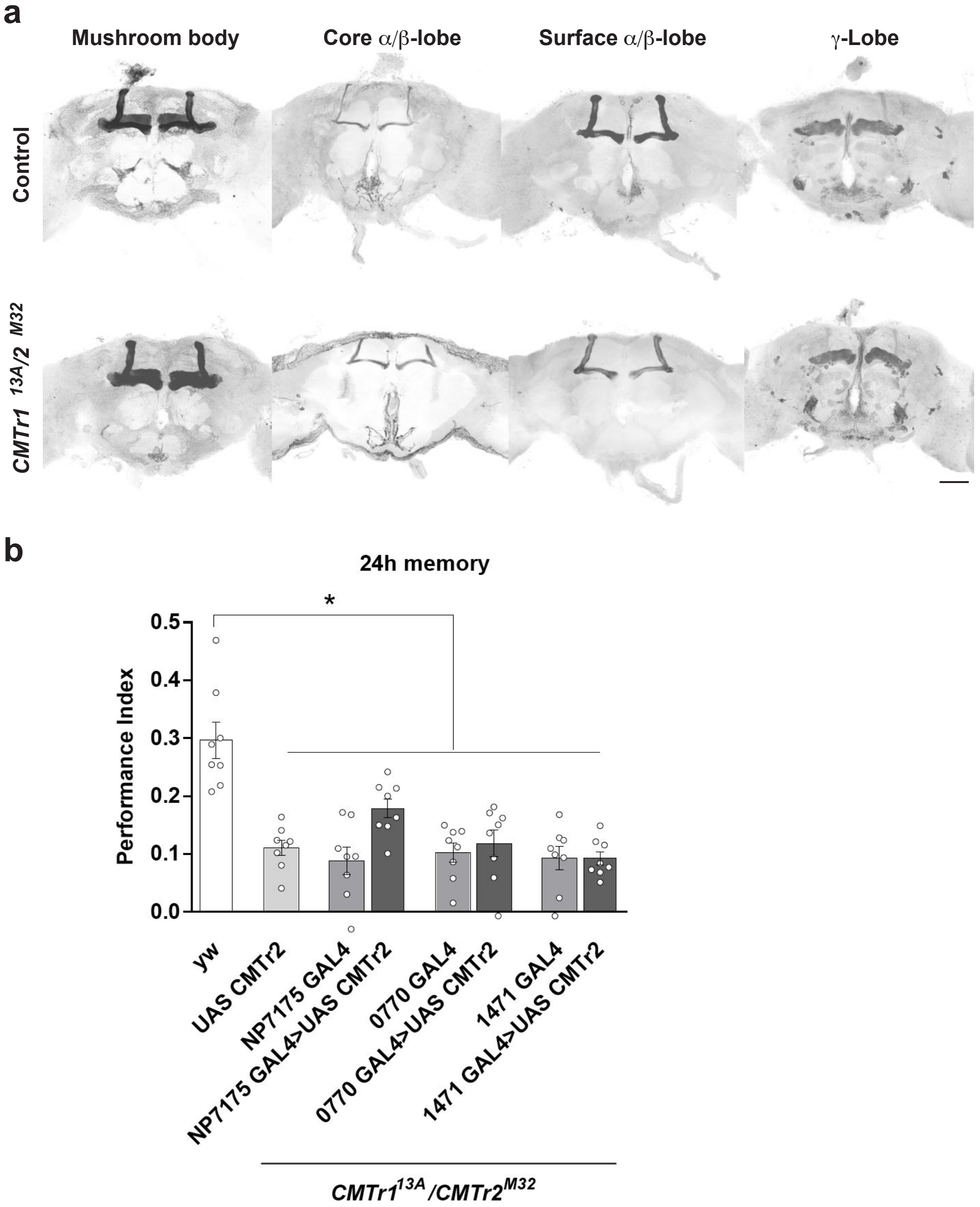

Supplementary Figure 5. mRNA cap-adjacent 2`-*O*-ribose methylation does not affect mushroom body morphology and is broadly required in the mushroom bodies for learning.

(a) Mushroom body morphology visualized by EGFP expression from *UAS* driven by *MB247-GAL4* (entire mushroom body), *NP7175-GAL4* (core alpha/beta lobe), *0770-GAL4* (surface alpha/beta lobe) and *1471- GAL4* (gamma lobe), respectively shown for control flies (upper row) and *CMTr1*^{13A}; *CMTr2*^{M32} double mutant flies. The scale bars is 100 µm. **(b)** Appetitive memory 24 hour after training of control flies compared to *CMTr1*^{13A}; *CMTr2*^{M32} double mutant flies expressing CMTr2 from *UAS* of the indicated *GAL4* drivers shown as mean±SE. n=8, p<0.0001. Statistical analysis was done by one way ANOVA followed by Dunnett's multiple comparison test.

Source data for memory experiments are provided as a Source Data file.

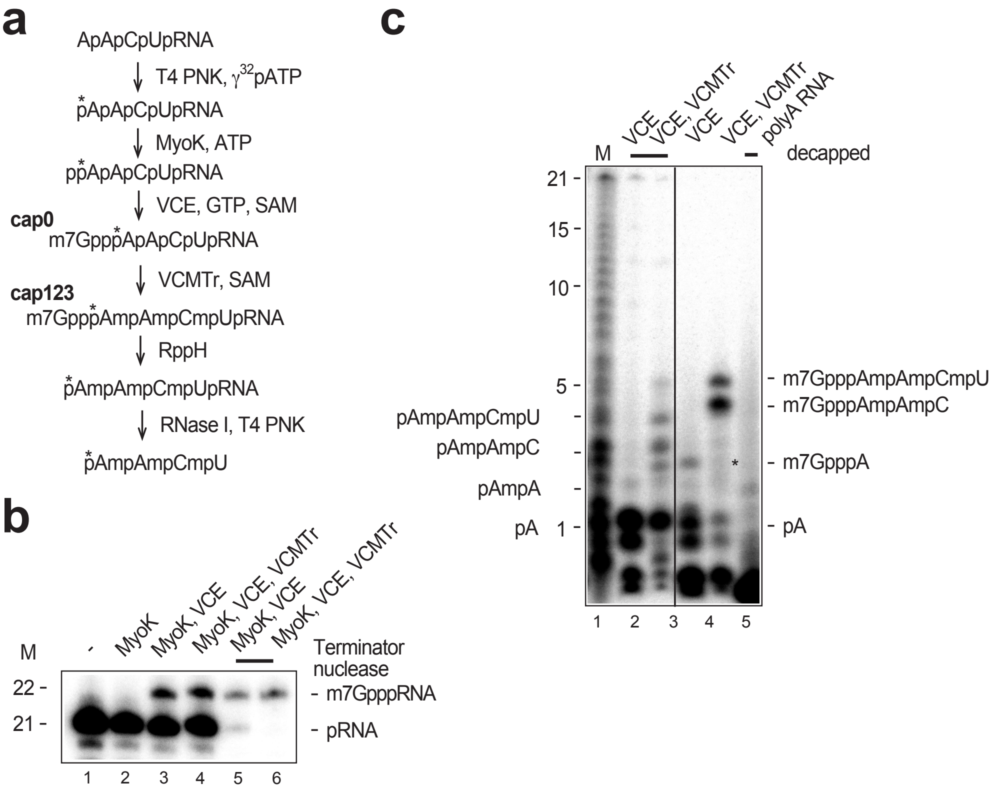

Supplementary Figure 6. Generation and analysis of capped RNA oligonucleotide with 2`-*O*-ribose methylated cap-adjacent nucleotides.
(a) Scheme for labeling of an RNA probe on the first adenosine as described in the methods section. **(b)** Representative gel from two replicates showing removal of uncapped RNA probe by terminator nuclease. M: length in nucleotides. **(c)** Representative gel from two replicates showing 2`-*O*-ribose methylation by vaccinia CMTr after digestion of the RNA probe with RNase I before and after decapping by RppH (line on top). The asterisk indicates the position of the m7GpppA cap, which is absent after RNAse I digestion when the substrate was 2`-*O*-ribose methylated with VCMTr. VCE: Vaccinia capping enzyme, VCMTr: vaccinia 2`-*O*-ribose methyltransferase. M: single nucleotide ladder.
Source data for gels are provided as a Source Data file.

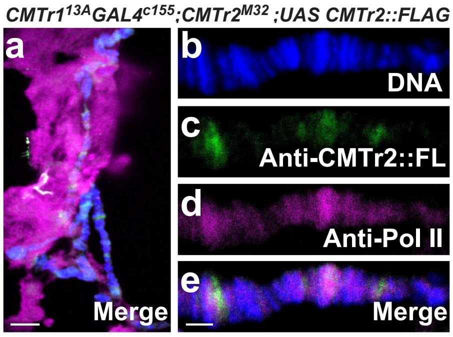

Supplementary Figure 7. CMTr2 does not re-localize to other targets in the absence of CMTr1. (**a-e**) Representative images of polytene chromosomes from salivary glands from three replicates expressing CMTr2::FLAG (a-e) in *CMTr1*^{13A}; *CMTr2*^{M32} double mutant flies stained with anti-Pol II (magenta, d), anti-FLAG (green, c) and DNA (DAPI, blue, b), or merged (white, a and e). Arrow heads indicate absence of CMTr2. Scale bars in a are 10 µm and in e are 2 µm.

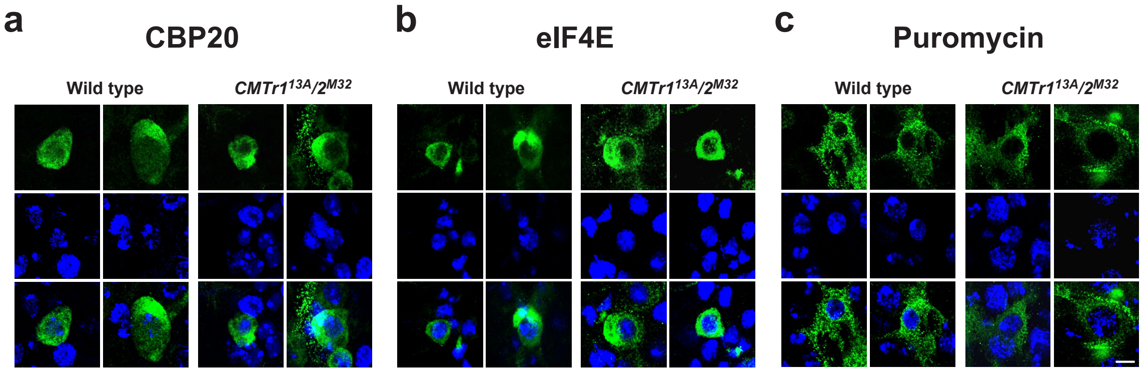

Supplementary Figure 8. Cellular localization of CBP20 and eIF4e, and puromycin incorporation in neurons of the ventral nerve cord.
(a-c) Staining of representative neurons in the ventral nerve cord of third instar larvae from five replicates each expressing HA epitope-tagged CBP20 (a) and eIF4e (b) with $elav^{C155}$-*GAL4* from *UAS* and puromycin incorporation (c) detected with an anti-puromycin antibody (top, green) and counter stained with DAPI (blue) in wild type and $CMTr1^{13A}$; $CMTr2^{M32}$ double mutant larvae. The scale bar in c is 2 µm.
