# Supplementary material for: CMTr cap-adjacent 2′-O-ribose mRNA methyltransferases are required for reward learning and mRNA localization to synapses
Source: Nat Commun. 2022 Mar 8;13:1209. doi: 10.1038/s41467-022-28549-5 (PMC8904806; doi:10.1038/s41467-022-28549-5)
Supplement: Supplementary file 3 — Description of Additional Supplementary Files [file 41467_2022_28549_MOESM3_ESM.pdf]

Description of Additional Supplementary Files

File Name: Supplementary Data 1
Description: Differential gene expression data.

File Name: Supplementary Data 2
Description: Functional analysis of differentially expressed genes.

File Name: Supplementary Data 3
Description: CMTr1 CLIP targets.

File Name: Supplementary Data 4
Description: CMTr2 CLIP targets.

File Name: Supplementary Data 5
Description: Overlap between CMTr and FMRP CLIP targets.
