# Supplementary material for: CMTr cap-adjacent 2′-O-ribose mRNA methyltransferases are required for reward learning and mRNA localization to synapses
Source: Nat Commun. 2022 Mar 8;13:1209. doi: 10.1038/s41467-022-28549-5 (PMC8904806; doi:10.1038/s41467-022-28549-5)
Supplement: Supplementary file 9 — Reporting Summary [file 41467_2022_28549_MOESM9_ESM.pdf]

nature research

Corresponding author(s): Matthias Soller
m.soller@bham.ac.uk

Last updated by author(s): Dec 21., 2021

Reporting Summary

Nature Research wishes to improve the reproducibility of the work that we publish. This form provides structure for consistency and transparency in reporting. For further information on Nature Research policies, see our Editorial Policies and the Editorial Policy Checklist.

Statistics

For all statistical analyses, confirm that the following items are present in the figure legend, table legend, main text, or Methods section.

n/a	Confirmed	
☐	☒	The exact sample size (*n*) for each experimental group/condition, given as a discrete number and unit of measurement
☐	☒	A statement on whether measurements were taken from distinct samples or whether the same sample was measured repeatedly
☐	☒	The statistical test(s) used AND whether they are one- or two-sided<br>*Only common tests should be described solely by name; describe more complex techniques in the Methods section.*
☒	☐	A description of all covariates tested
☐	☒	A description of any assumptions or corrections, such as tests of normality and adjustment for multiple comparisons
☐	☒	A full description of the statistical parameters including central tendency (e.g. means) or other basic estimates (e.g. regression coefficient) AND variation (e.g. standard deviation) or associated estimates of uncertainty (e.g. confidence intervals)
☐	☒	For null hypothesis testing, the test statistic (e.g. *F*, *t*, *r*) with confidence intervals, effect sizes, degrees of freedom and *P* value noted<br>*Give P values as exact values whenever suitable.*
☒	☐	For Bayesian analysis, information on the choice of priors and Markov chain Monte Carlo settings
☒	☐	For hierarchical and complex designs, identification of the appropriate level for tests and full reporting of outcomes
☒	☐	Estimates of effect sizes (e.g. Cohen's *d*, Pearson's *r*), indicating how they were calculated

Our web collection on statistics for biologists contains articles on many of the points above.

Software and code

Policy information about availability of computer code

Data collection	Leica TCS SP8 Las-X
Data analysis	GraphPad Prism (v6 & v7 as indicated), Fiji Image J 1.53c, QuantityOne (Biorad), EnhancedVolcano Version 1.4.0, ggplot2, DESEQ2, STAR 2.7.9a

For manuscripts utilizing custom algorithms or software that are central to the research but not yet described in published literature, software must be made available to editors and reviewers. We strongly encourage code deposition in a community repository (e.g. GitHub). See the Nature Research guidelines for submitting code & software for further information.

Data

Policy information about availability of data

All manuscripts must include a data availability statement. This statement should provide the following information, where applicable:

- Accession codes, unique identifiers, or web links for publicly available datasets
- A list of figures that have associated raw data
- A description of any restrictions on data availability

All data are available in the main text or the supplementary material and gene expression data have been deposited at GEO under the accession numbers GSE116212, GSE181321 and GSE138868. Source data are provided with this paper as a Source Data file.

Field-specific reporting

Please select the one below that is the best fit for your research. If you are not sure, read the appropriate sections before making your selection.

☒ Life sciences ☐ Behavioural & social sciences ☐ Ecological, evolutionary & environmental sciences

For a reference copy of the document with all sections, see nature.com/documents/nr-reporting-summary-flat.pdf

Life sciences study design

All studies must disclose on these points even when the disclosure is negative.

Sample size	No statistical methods were used to predetermine sample size. Sample sizes were chosen according to typical sizes in the field.
Data exclusions	No data were excluded.
Replication	All replicates were successful.
Randomization	The experiments were not randomized and samples were allocated according to genotypes.
Blinding	Investigators were not blinded to allocation during experiments and outcome assessment.

Reporting for specific materials, systems and methods

We require information from authors about some types of materials, experimental systems and methods used in many studies. Here, indicate whether each material, system or method listed is relevant to your study. If you are not sure if a list item applies to your research, read the appropriate section before selecting a response.

Materials & experimental systems

n/a	Involved in the study	
☐	☒	Antibodies
☐	☒	Eukaryotic cell lines
☒	☐	Palaeontology and archaeology
☐	☒	Animals and other organisms
☒	☐	Human research participants
☒	☐	Clinical data
☒	☐	Dual use research of concern

Methods

n/a	Involved in the study	
☒	☐	ChIP-seq
☒	☐	Flow cytometry
☒	☐	MRI-based neuroimaging

Antibodies

Antibodies used

Antibodies used in this study are described in the Methods section.
Rat monoclonal anti-HA 3F10 (Roche Cat# 11867423001)
Rabbit monoclonal anti-FLAG M2 (Sigma Cat# F2555)
Mouse anti-Pol II H5 IgM (Abcam Ab24758)
Mouse monoclonal anti-NC82 (DSHB Cat# NC82)
Mouse monoclonal anti-ELAV 7D (gift from White, K.(Haussmann et al., 2008)
Rabbit polyclonal anti-CBP80 (gift from Kopytova, D. (Gurskiy et al., 2012)
Anti-Puromycin Antibody, clone 12D10 (Merck Cat# MABE343)
Mouse Anti-tubulin (Sigma Cat# T5186)
Mouse Monoclonal ANTI-FLAG® M2 (Sigma Cat# 3165)
DAPI (Sigma Cat# D9542)
Goat anti-Rat IgG (H+L) Alexa Fluro 488 (Thermo Fischer Scientific Cat# A-11006, RRID: AB_2534074)
Goat anti-Rabbit IgG (H+L) Alexa Fluro 488 (Thermo Fischer Scientific Cat# A-11008, RRID: AB_143165)
Goat anti-Mouse IgG (H+L) Alexa Fluro 633 (Thermo Fischer Scientific Cat# A-21052, RRID: AB_2535719)
Goat anti-Mouse IgM (H+L) Alexa Fluro 488 (Thermo Fischer Scientific Cat# A-10680) and Goat Anti-Mouse IgG Antibody, HRP conjugate (Sigma Cat# 12-349)

Validation

Antibodies used were validated by in situ staining or Western blots.

Eukaryotic cell lines

Policy information about cell lines

Cell line source(s)	Drosophila S2 cells, ATTC
Authentication	S2 cells are male
Mycoplasma contamination	cells for cell extracts were not tested for mycoplasma
Commonly misidentified lines (See ICLAC register)	no commonly misidentified cell lines were used.

Animals and other organisms

Policy information about studies involving animals; ARRIVE guidelines recommended for reporting animal research

Laboratory animals	Drosophila melanogaster were used in this study. Larval and adult stages were used, and genotypes are given in the Methods section and/or the text.
Wild animals	none
Field-collected samples	none
Ethics oversight	none

Note that full information on the approval of the study protocol must also be provided in the manuscript.
